# One pot synthesis, antimicrobial and antioxidant activities of fused uracils: pyrimidodiazepines, lumazines, triazolouracil and xanthines

**DOI:** 10.1186/s13065-017-0294-0

**Published:** 2017-07-19

**Authors:** Samar A. El-Kalyoubi, Eman A. Fayed, Ahmed S. Abdel-Razek

**Affiliations:** 1 Department of Pharmaceutical Organic Chemistry, Faculty of Pharmacy (Girls), Al-Azhar University, Nasr City, Cairo, 11651 Egypt; 2 Medical Chemistry Department, Faculty of Medicine (Female Section), Jazan University, Jazan, 45142 Saudi Arabia; 3 Department of Microbial Chemistry, Genetic Engineering and Biotechnology Division, National Research Centre, El-Buhouth St., Dokki-Cairo, 12622 Egypt

**Keywords:** 5,6-diaminouracil, Pyrimidodiazepine, Lumazine, Xanthine, Triazolouracil, Antimicrobial and antioxidant activities

## Abstract

**Background:**

Uracil derivatives have a great attraction because they play an important role in pharmacological activities. Pyrimidodiazepines, lumazines, triazolopyrimidines and xanthines have significant wide spectrum activities including anticancer, antiviral as well as antimicrobial activities.

**Results:**

A newly synthesized compounds pyrimido[4,5-*b*][1, 4]diazepines **5a**–**e**, **6a**–**d**, lumazines **7a**–**d**, triazolo[4,5-*d*]pyrimidine **8** and xanthines **9**, **10** was prepared in a good yields. The antimicrobial and antioxidant activities of compounds **5a**, **5b**, **6a**, **6d** and **8** exhibited a wide range activity against the pathogenic tested microbes (*Staphylococcus aureus*, *Bacillus subtilis*, *Pseudomonas aeruginosa*, *Candida albicans*, and *Saccharomyces cerevisiae*). Compound **8** showed activity against the fungus *Aspergillus niger*. The highest antioxidant activity was noticed for compound **5a**.

**Conclusions:**

A series of novel pyrimido[4,5-*b*][1, 4]diazepines **5a**–**e**,** 6a**–**d**, lumazines **7a**–**d**, triazolo[4,5-*d*]pyrimidine **8** and xanthines **9**, **10** was prepared from 5,6-diamino-1-(2-chlorobenzyl)uracil **3** in good yields. Compounds **5a**–**e**,** 6a**–**d** were prepared by sequential manipulation of **3** with *α*,*β*-unsaturated ketones. Lumazines **7a**–**d** were obtained from **3** by treatment with phenacyl bromides in the presence of TEA. Compound **8** was prepared by treatment of **3** with HNO_2_, while xanthines **9**, **10** were obtained from **3** by consecutive acetylation then intramolecular cyclodehydration or heating with malononitrile under solvent-free condition. The antimicrobial and antioxidant activity of this series was evaluated in vitro and they showed either weak or moderate activities.

**Graphical abstract:**
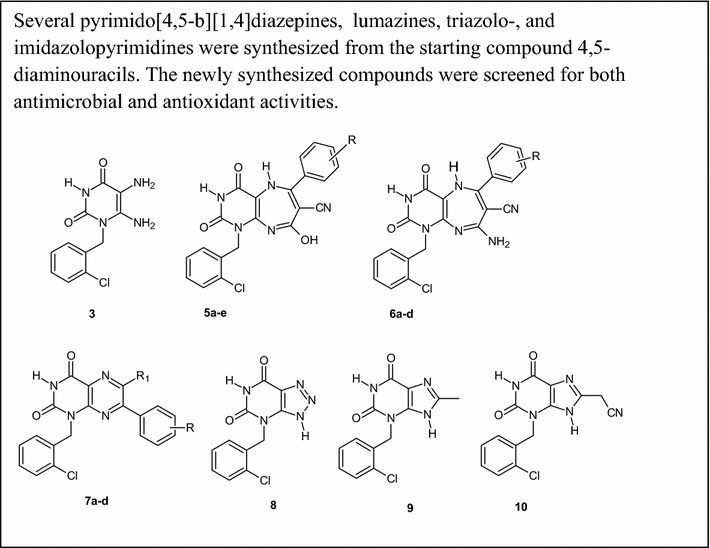
Several pyrimido[4,5-*b*][1,4]diazepines, lumazines, triazolo-, and imidazolopyrimidines were synthesized from the starting compound 4,5-diaminouracils. The newly synthesized compounds were screened for both antimicrobial and antioxidant activities.

## Background

Uracil is a basic scaffold for design of significant pharmaceuticals [1–6]. They displayed wide spectrum activities including anticancer [7–12], antiviral [13–19] and antimicrobial activities [20–25]. Bacterial infections continue to represent a major worldwide health problem. Many pathogenic bacteria have resistance to antibacterial agents through a variety of mechanisms. Ironically, the drug-resistant strains became widespread due to the misuse of antibiotics. This arsenal of drug-resistant strains is resistant to most available antibiotics [26–28], thus lead to severe morbidity and mortality of the patients.

To solve these problems, researchers are required to modify the structure of uracil and subsequently these problems can be overcome by innovation of new derivatives with beneficial pharmacological and pharmacokinetic effects. These new fused uracil derivatives as antibacterial agents can be obtained via replacement at N-1, N-3, C-5 and C-6 positions with different substituents on uracil ring. Seven-member heterocyclic compounds containing nitrogen atom, such as 1,4-diazepine derivatives, are considered as an important drug discovery because they have a wide range of antimicrobial activities [29].

The purpose of this study is to evaluate the in vitro effect of antimicrobial fused uracil derivatives, pyrimidodiazepines, lumazines, triazolouracil and xanthines. Simultaneously, a MIC-kinetic curve for the inhibition activity of the new molecules was also obtained. The structure of newly synthesized uracil-based derivatives was proven on the basis of their ^1^H-NMR, mass spectral data, IR and elemental analysis.

## Results and discussion

### Chemistry

**Scheme 1:**
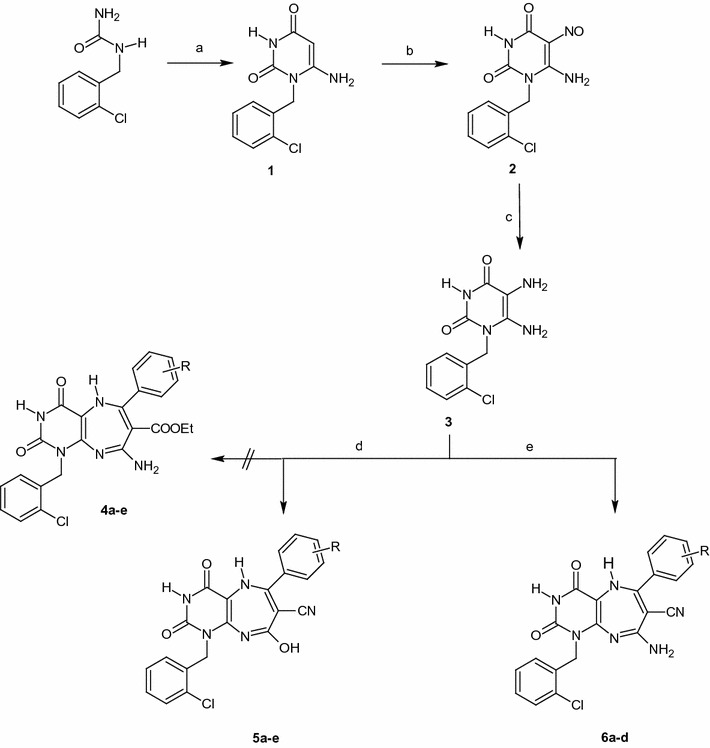
Reagents and conditions: *a* NCCH_2_COOEt, EtONa, Reflux; *b* NaNO_2_, HCl, rt; *c* (NH_4_)_2_S; *d* ArCH=C(CN)COOEt, TEA, DMF, Reflux. [**5a** (R=H, 66%); **5b** (R=4−Cl, 57%); **5c** (R=4−Br, 57%); **5d** (R=2−OH, 51%); **5e** (R = 3−NO_2_, 49%)]; *e* ArCH=C(CN)_2_, TEA, DMF, Reflux. [**6a** (R=H, 53%); **6b** (R=4−Cl, 69%); **6c** (R=4−Br, 64%); **6d** (R=2−OH, 54%)]

To our endeavor toward developing new uracil-based architectures of potential pharmacological significance, 5,6-diamino-1-(2-chlorobenzyl)uracil **3** [30] was chosen as scaffold for annulations of the target congeners. This substrate was prepared from 1-(2-chlorobenzyl)urea by consecutive cyclization with ethylcyanoacetate in the presence of sodium ethoxide [31–33], nitrosation with in situ prepared HNO_2_ [30, 34] then reduction with (NH_4_)_2_S [30] (Scheme 1). Series **5a**–**e** was prepared in moderate yield (49–66%) by refluxing compound **3** with different arylidene ethylcyanoacetates in DMF containing TEA for 6–7 h. All derivatives were recrystallized from DMF/EtOH. The reaction proceeded through Michael addition reaction via the formation of non-isolated Michael adduct intermediate that undergo cyclocondensation accompanied by elimination of EtOH followed by oxidation affording the corresponding 1-(2-chlorobenzyl)-8-hydroxy-6-(aryl)-2,4-dioxo-2,3,4,5-tetrahydro-1*H*-pyrimido[4,5-*b*][1, 4]diazepine-7-carbonitrile. The IR spectra of these diazepines displayed the C≡N stretching band at 2222–2217 cm^−1^ confirming cyclization, the stretching band of the two C=O groups (*Amide I*) was red-shifted within the range 1690–1610 cm^−1^. Derivatives **5d**, **e** displayed two separate bands for the two C=O groups. The imide linkages in this series displayed keto-iminol tautomerism, since they showed O–H stretching bands 3634–3617 cm^−1^ and additional O–H stretching bands in compound **5d** at 3495 cm^−1^ and N–H stretching bands 3164–3141 cm^−1^. The nitro group in compound **5e** showed strong asymmetric and symmetric NO_2_ stretching bands at 1518 and 1350 cm^−1^, respectively. The intrinsic significance of the IR spectra is that they exclude the possibility of the cyclization pass way that lead to compounds **4a**–**e** due to absence of any blue-shifted C=O stretching bands.

The ^1^H-NMR spectra supported the previous observation from the IR spectra, where compounds **4a**–**e** are excluded, as the ethyl fingerprint signals were not observed. The singlet of the NCH_2_ protons (*δ* 5.25–5.23 ppm) were the most shielded as expected, while the C8-OH and N3–H were highly deshielded. They appeared around *δ* 14.0 and 11.4 ppm, due to flanking of the N3–H between the two C=O groups and strong magnet anisotropic effect of the imine linkage on C8-OH group. Thus, the N5–H signal is most likely to be overlapped with the signals of the aromatic protons. The downfield shift of the C=O groups in the ^13^C-NMR spectra, for instance **5b**, is typical for imides as sequel of bond order reduction by keto-iminol tautomerism or overlap of the nitrogen’s lone-pair of electrons with the π-cloud of the C=O group.

**Scheme 2:**
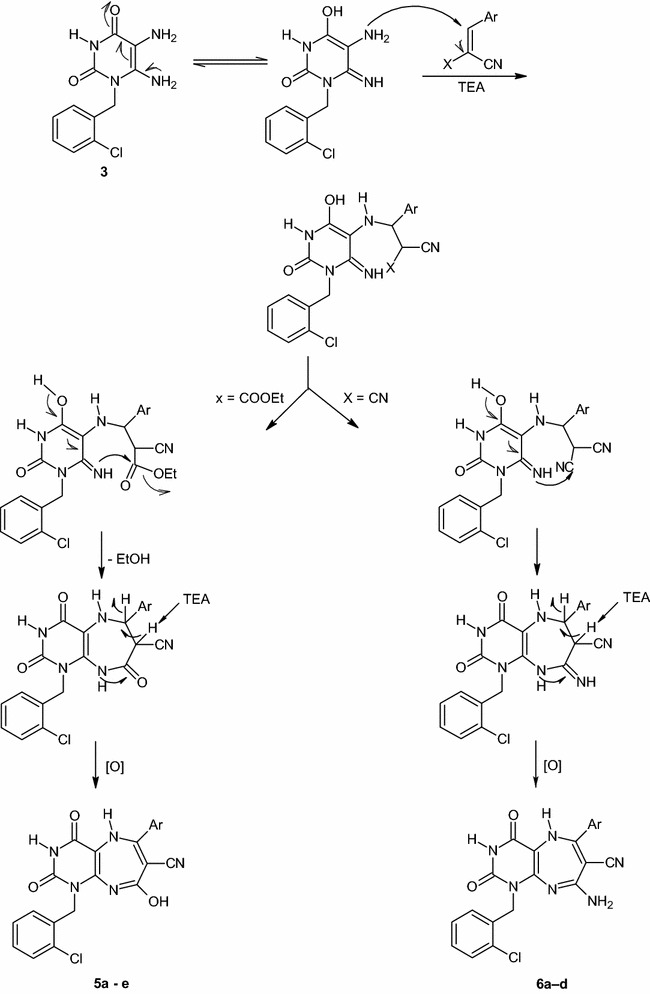
Plausible mechanism for the formation of compounds **5a**–**e** and **6a**–**d**

Refluxing of **3** with different arylidenemalononitriles in refluxing DMF containing TEA afforded the corresponding 8-Amino-1-aryl-6-(4-chlorophenyl)-2,4-dioxo-2,3,4,5-tetrahydro-1*H*-pyrimido[4,5-*b*][1, 4]diazepine-7-carbonitriles **(6a**–**d)** in 53–69% yields after recrystallization from DMF/EtOH (Scheme 1). The reaction proceeded exactly as for compounds **5a**–**e**; Michael addition then cycloaddition on one nitrile group as unique possible lane. The IR spectra were in accordance with the proposed structures and the common bands with compounds **5a**–**e** were within similar frequencies ranges. The most interesting conclusion from comparing the ^1^H-NMR spectra of these derivatives with compounds **5a**–**e** is the absence of the signal at *δ* 14.36–13.98 ppm in compounds **6a**–**d**. This confirms without doubt that this signal is attributed to the C8-OH group in compounds **5a**–**e**, the group that does not exist in compounds **6a**–**d**. The signals at *δ* 7.77–7.54 ppm are believed to be for the C8-NH_2_ protons. A reasonable mechanism for this reaction is shown in (Scheme 2).

**Scheme 3:**
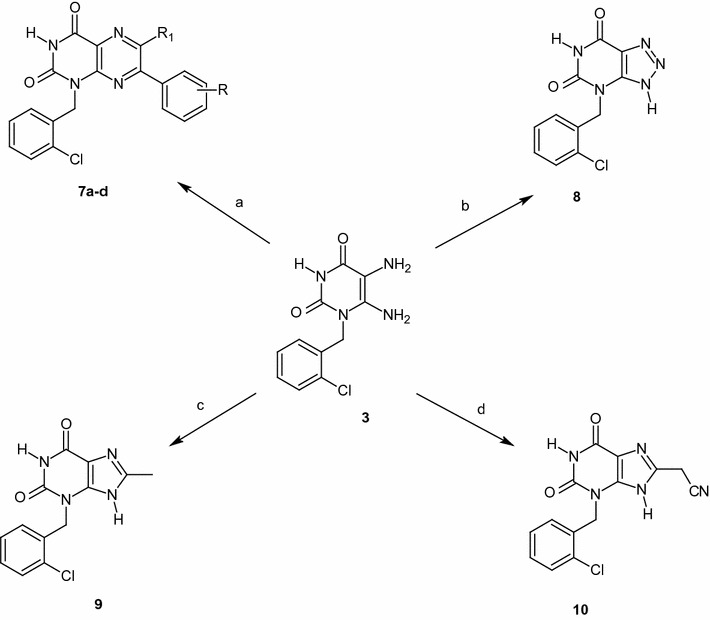
Reagents and conditions*: a* ArCOCH(R_1_)Br, TEA, DMF, Reflux. [**7a** (R=R_1_=H (71%); **7b** (R = 4−OMe, R_1_=H (74%); **7c** (R=H, R_1_=ph (68%);** 7d** (R=4−NO_2_, R_1_ = H (58%)]; *b* NaNO_2_, HCl, rt, **8** (78%); *c* Ac_2_O, AcOH, Reflux, **9** (72%); *d* CH_2_(CN)_2_, heating under solvent-free condition, **10** (77%)

Pteridine is a basic component of folic acid, bacteria use it as starting material for its own multi stage tetrahydrofolic acid`s (FH_4_) biosynthesis and, consequently the production of nucleic acid bases necessary for its replication. Sulphonamides (sulpha drugs) are common inhibitors of FH_4_ biosynthesis and act as bacteriostatic. Therefore, substrate **3** was treated with different phenacyl bromides in refluxing DMF containing TEA to afford lumazines **7a**–**d** in good yields as potential folate antagonists (Scheme 3).

**Scheme 4:**
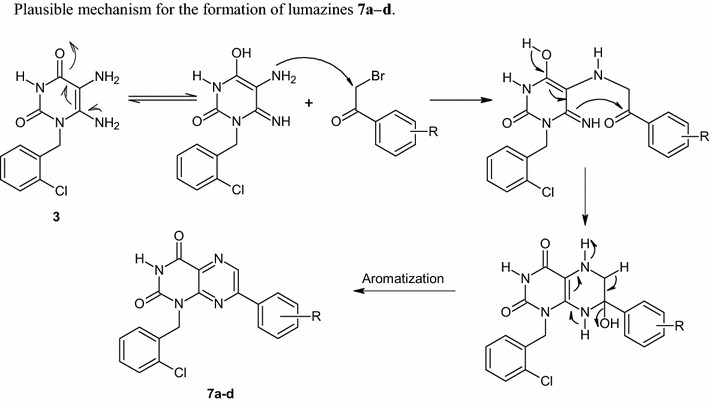
Plausible mechanism for the formation of pteridines **7a−d**

Formation of lumazines **7a**–**d,** presumably proceeded via S_N_2 alkylation of C5-NH_2_ followed by aromatization through synchronous dehydration and oxidation steps (Scheme 4).

The IR spectra of this series showed the N–H stretching bands within the range 3174–3100 cm^−1^. The two C=O groups gave rise to two bands ≈1725 and ≈1680 cm^−1^. Pteridine **7d** displayed the two characteristic bands of the NO_2_ group at 1515, 1368 cm^−1^.

The ^1^H-NMR spectra of compounds **7a**, **b** and **d** showed characteristic singlet for the N–H protons at *δ* 12.15–12.00 ppm and a singlet at *δ* 9.32–9.14 ppm for H-6. Compound **7b** showed a signal at *δ* 3.82 ppm for the methyl group, besides the CH_2_ signal at *δ* 5.44 ppm. The shift of the CH_3_ signal was observed at *δ* 42.2 ppm in the ^13^C-NMR spectrum.

Triazolopyrimidine **8** was prepared in good yield by cyclocondensation of substrate **3** with in situ prepared HNO_2_ at ambient temperature. The triazole’s N–H signal was abnormally observed highly deshielded at *δ* 15.76 ppm, beside the pyrimidine N3-H at *δ* 11.61 ppm. The shift of the CH_2_ carbon was observed normally at *δ* ≈44.30 ppm in the ^13^C-NMR spectrum.

Xanthine **9** was prepared in 72% yield by refluxing of substrate **3** with Ac_2_O in AcOH. The ^1^H-NMR spectrum showed characteristic two broad singlets for the 2N–H protons at *δ* 13.19 and 11.15 ppm. The CH_3_ signal appeared upfiled at *δ* 2.31 ppm and its carbon appeared at *δ* 14.20 ppm in the ^13^C-NMR spectrum. Surrogate **10** was prepared in 77% yield from compound **3** by heating with CH_2_(CN)_2_ under solvent-free condition. The IR spectrum displayed the C≡N stretching band at proper frequency 2200 cm^−1^, while the ^1^H-NMR disclosed two signals at *δ* 5.08 ppm for the NCH_2_ protons and at *δ* 4.10 ppm for the protons in the CH_2_CN group.

This series displayed, in their EI-MS spectra, molecular ions peaks corresponding to the mass of each formula and their elemental analyses agreed as well.

### Biological activity

#### Antimicrobial activity

Antimicrobial activity assay results (Table 1) revealed that compound **6b** exhibited low to moderate activity only against *Pseudomonas aeruginosa*. Compound **7a** exhibited low to moderate activity only against *Saccharomyces cerevisiae*. Some other compounds (**5a**, **5b**, **6a**, **6d** and **8**) exhibited activities against wide range of pathogenic tested microbes. The minimal inhibitory concentrations (MIC) of these compounds had been measured (Table 2). MIC is the lowest concentration of substance that inhibits the growth of microorganism.

**Table 1:** *In vitro* antimicrobial activity of compounds **5−10** expressed as inhibition zone diameters (mm)

Code	*S. aureus*		*B. subtilis*		*P. aeruginosa*		*C. albicans*		*S. cerevisiae*		*A. niger*	
	*A*	*B*	*A*	*B*	*A*	*B*	*A*	*B*	*A*	*B*	*A*	*B*
**5a**	8	9	9	10	10	9	12	11	9	10	−	−
**5b**	8	9	8	10	9	14	9	10	8	12	−	−
**5c**	−	−	−	−	−	−	−	−	−	−	−	−
**5d**	−	−	−	−	−	−	−	−	−	8	−	−
**5e**	−	−	−	−	−	−	−	−	−	−	−	−
**6a**	10	11	11	10	9	9	10	10	−	−	−	−
**6b**	−	−	−	−	8	10	−	−	−	−	−	−
**6c**	−	−	−	−	−	−	−	−	−	−	−	−
**6d**	10	14	13	10	11	12	12	12	10	15	−	−
**7a**	−	−	−	−	−	−	−	−	9	10	−	−
**7b**	−	−	−	−	−	−	−	−	−	−	−	−
**7c**	−	−	−	−	−	−	−	−	−	−	−	−
**8**	10	11	−	−	10	12	−	−	12	12	10	9
**9**	−	−	−	−	−	−	−	−	−	−	−	−
**10**	−	−	−	−	−	−	−	−	−	−	−	−
**Bc**	10	11	8	10	18	15	−	−	−	−	−	−
**Fc**	−	−	−	−	−	−	12	14	−	−	20	18

*A* Paper−disk method and *B* well-agar method using 20 µl of 50 mg/ml of test compounds

*Bc* antibacterial positive control, *Fc* is (positive antifungal control), *−* no activity

**Table 2:** MIC values in ppm of compounds **5a**, **5b**, **6a**, **6d** and **8**

	*S. aureus*	*P. aeruginosa*	*B. subtilis*	*C. albicans*	*S. cerevisiae*	*A. niger*
**5a**	5.0 × 10^−3^	5.0 × 10^−4^	5.0 × 10^−3^	5	5	−
**5b**	0.5	0.5	5	5	5	–
**6a**	0.12	12.5	1.25	12.5	–	–
**6d**	0.5	5.0 × 10^−4^	5.0 × 10^−2^	0.5	5.0 × 10^−2^	–
**8**	0.5	5.0 × 10^−4^	5	5	0.5	0.5

− not measured

**Fig. 1:**
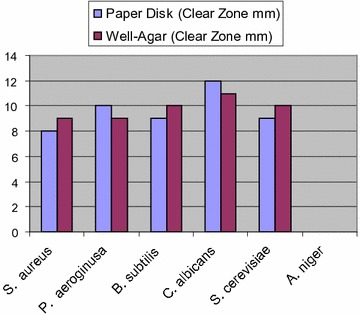
Antimicrobial activity of compound **5a** using agar disk diffusion method

Compound **5a** exhibited low activity against *Staphylococcus aureus*; low to moderate activity against *P. aeruginosa, Bacillus subtilis* and *S. cerevisiae*, but showed moderate to strong activities against *Candida albicans *(Fig. **1**).

**Fig. 2:**
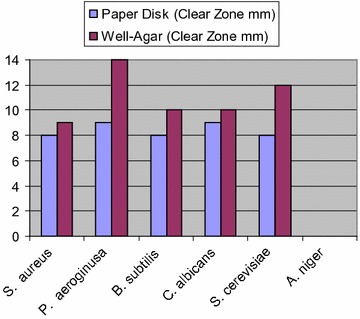
Antimicrobial activity of compound **5b** using agar disk diffusion method

Compound **5b** exhibited low to moderate activity against *S. aureus*, *B. subtilis* and *C. albicans*, but showed moderate to strong activities against *P. aeruginosa* and *S. cerevisiae* (Fig. 2).

**Fig. 3:**
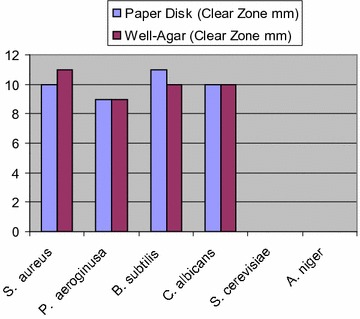
Antimicrobial activity of compound **6a** using agar disk diffusion method

Compound **6a** exhibited moderate activity against *S. aureus*, *B. subtilis* and *C. albicans*, but showed low activity against *P. aeruginosa*, and showed no activity against *S. cerevisiae* and *Aspergillus niger* (Fig. 3).

**Fig. 4:**
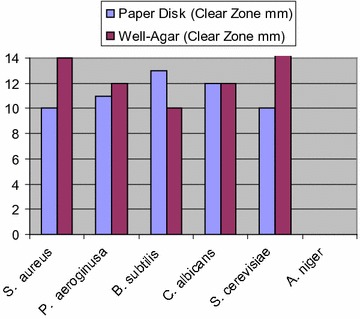
Antimicrobial activity of compound **6d** using agar disk diffusion method

Compound **6d** exhibited moderate to strong activity against all test microbes except for the fungus *A. niger* (Fig. 4).

**Fig. 5:**
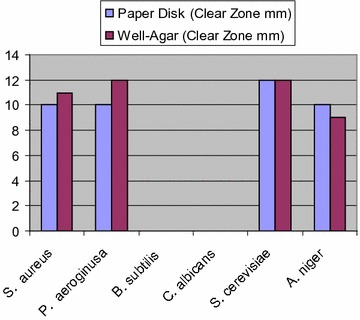
Antimicrobial activity of compound **8** using agar disk diffusion method

Compound **8** was the only compound that exhibited activity against the fungus *A. niger*. Also, it exhibited moderate activity against *S. aureus*; strong activity against *S. cerevisiae*, and moderate to strong activity against *P*. *aeroginosa* but showed no activity against *B. subtilis* and *C. albicans* (Fig. 5).

#### Antioxidant activity

The percentages of antioxidant activity (AA%) of compounds **(5a**–**e, 6a**–**d, 7a**–**c** and **8**–**10)** have been measured (Table 3) and the results revealed that the compound **5a** showed the highest activity (39.9%) followed by the compound **8**. The lowest antioxidant activity recorded for the compound **6c** is 1.9. Two compounds **7a** and **7b** showed no antioxidant activity.

**Table 3:** The percentage of antioxidant activity (AA%) for the samples (**5a**–**e, 6a**–**d, 7a**–**c** and **8**–**10**)

Sample code	5a	5b	5c	5d	5e	6a	6b	6c	6d	7a	7b	7c	8	9	10
AA%	39.9	27	11	2.8	3.6	2.9	5.5	1.9	9	0	0	2.6	29.1	3.8	22.9

## Experimental section

Materials and instruments

All melting points were determined by an Electrothermal Mel.-Temp. II apparatus and were uncorrected. Element analyses were performed at Regional Center for Mycology and Biotechnology at Al-Azhar University. The infrared (IR) spectra were recorded using potassium bromide disc technique on Nikolet IR 200 FT IR. Mass spectra were recorded on DI-50 unit of Shimadzu GC/MS-QP 5050A at the Regional Center for Mycology and Biotechnology at Al-Azhar University. The proton nuclear magnetic resonance (^1^H-NMR) spectra were recorded on Bruker 400 MHz Spectrometer and ^13^C-NMR spectra were run at 125 MHz in dimethylsulfoxide (DMSO-*d*6) and TMS as an internal standard, Applied Nucleic Acid Research Center, Zagazig University, Egypt. All new compounds gave corresponding elemental analyses (C, H, N, typically ±0.3%). All reactions were monitored by TLC using precoated plastic sheets silica gel (Merck 60 F_254_) and spots were visualized by irradiation with UV light (254 nm). The used solvent system was chloroform: methanol (9:1) and ethyl acetate: toluene (1:1).

### Synthetic procedures

#### *6*-*Amino*-*1*-*(2*-*chlorobenzyl)uracil* (**1**)

This compound was prepared according to a reported method [31–33], yield 68%, m.p. 295 °C.

#### *6*-*Amino*-*1*-*(2*-*chlorobenzyl)*-*5*-*nitrosouracil* (**2**)

This compound was prepared according to a reported method [30, 34], yield 95%, m.p. 236 °C [lit 235 °C].

#### *5,6*-*diamino*-*1*-*(2*-*chlorobenzyl)uracil* (**3**)

Compound **2** (6.0 g, 24.36 mmol) was added over 15 min to ammonium sulphide solution (36 ml) at 70–80 °C with stirring. The formed precipitate was collected by filtration, washed with ethanol and dried in vacuum desiccator to give 92% [30]. m.p. = 245–247 °C.

#### *6*-*Aryl*-*1*-*(2*-*chlorobenzyl)*-*8*-*hydroxy*-*2,4*-*dioxo*-*2,3,4,5*-*tetrahydro*-*1H*-*pyrimido[4,5*-*b][1, 4]diazepine*-*7*-*carbonitriles* (**5a**–**e**)

A mixture of 5,6-diamino-1-(2-chlorobenzyl)uracil (**3**) (0.3 g, 1.12 mmol) and appropriate arylidene ethylcyanoacetate (1.12 mmol) in DMF (3 ml) in presence of drops of TEA was heated under reflux for 6–7 h. The reaction mixture was evaporated under reduced pressure. The residue obtained was suspended in ethanol, filtered and recrystallized from DMF/ethanol (2:1).

#### *1*-*(2*-*chlorobenzyl)*-*8*-*hydroxy*-*2,4*-*dioxo*-*6*-*phenyl*-*2,3,4,5*-*tetrahydro*-*1H*-*pyrimido[4,5*-*b][1, 4]diazepine*-*7*-*carbonitrile* (**5a**)

Yield: 66%, m.p. ≥ 300 °C. IR (ν_max_, cm^−1^) = 3634 (OH), 3164 (br, NH), 3026 (CH_arom_), 2812 (CH_aliph_), 2217 (CN), 1674 (C=O), 1550 (C=N), 1518 (C=C), 748 (*o*-substituted). MS: *m*/*z* (%) = 421 (M+2, 1.23), 419 (M^+^, 2.33), 261 (31), 257 (33), 255 (13), 184 (15), 183 (76), 171 (42), 168 (16), 124 (99), 121 (35), 95 (20), 81 (82), 55 (100), 45 (62). ^1^H-NMR (DMSO-d_6_) *δ* ppm: 14.03 (1H, s, OH, exchangeable), 11.35 (1H, s, NH, exchangeable), 7.99–7.95 (3H, m, NH, exchangeable and 2H_arom_), 7.68–7.66 (2H, d, *J* = 8.4 Hz, H_arom_), 7.52–7.50 (1H, d, *J* = 7.6 Hz, H_arom_), 7.32–7.25 (3H, m, H_arom_), 7.07–7.05 (d, 1H, *J* = 7.6 Hz, H_arom_), 5.23 (s, 2H, NCH_2_). Anal. Calcd for C_21_H_14_ClN_5_O_3_, Calcd.: C 60.08, H 3.36, N 16.68, Found C 60.21, H 3.39, N 16.84.

#### *1*-*(2*-*chlorobenzyl)*-*6*-*(4*-*chlorophenyl)*-*8*-*hydroxy*-*2,4*-*dioxo*-*2,3,4,5*-*tetrahydro*-*1H*-*pyrimido[4,5*-*b][1, 4]diazepine*-*7*-*carbonitrile* (**5b**)

Yield: 57%, m.p. ≥ 300 °C. IR (ν_max_, cm^−1^) = 3622 (OH), 3148 (br, NH), 3024 (CH_arom_), 2819 (CH_aliph_), 2221 (CN), 1683 (C=O), 1551 (C=N), 1520 (C=C), 834 (*p*-substituted), 749 (*o*-substituted). MS: *m*/*z* (%) = 458 (M + 4, 0.42), 456 (M + 2, 0.64), 454 (M^+^, 1.19), 397 (15), 395 (12), 289 (10), 259 (15), 241 (18), 236 (13), 213 (13), 183 (33), 182 (14), 149 (19), 110 (16), 107 (16), 97 (37), 96 (39), 95 (27), 94 (11), 86 (16), 85 (21), 84 (26), 82 (33), 72 (22), 71 (37), 70 (20), 69 (100), 68 (23), 57 (24), 45 (18). ^1^H-NMR (DMSO-d_6_) *δ* ppm: 14.01 (1H, s, OH), 11.36 (1H, s, NH), 8.06–8.04 (2H, d, *J* = 8.4 Hz, H_arom_), 7.55–7.50 (3H, m, NH & 2H_arom_), 7.30–7.23 (3H, m, H_arom_), 7.07–7.05 (1H, d, *J* = 7.2 Hz, H_arom_), 5.23 (2H, s, NCH_2_). ^13^C-NMR (DMSO-d_6_) *δ* ppm: 160.3, 159.0, 158.0, 154.7, 150.1, 135.8, 134.3, 133.1, 131.4, 129.5, 129.2, 128.4, 127.8, 127.3, 126.7, 151.1, 99.4, 88.6, 42.6. Anal. Calcd for C_21_H_13_Cl_2_N_5_O_3_, Calcd: C 55.52, H 2.88, N 15.42, Found: C 55.70, H 2.85, N 15.58.

#### *6*-*(4*-*bromophenyl)*-*1*-*(2*-*chlorobenzyl)*-*8*-*hydroxy*-*2,4*-*dioxo*-*2,3,4,5*-*tetrahydro*-*1H*-*pyrimido[4,5*-*b][1, 4]diazepine*-*7*-*carbonitrile* (**5c**)

Yield: 57%, m.p. ≥ 300 °C. IR (ν_max_, cm^−1^) = 3632 (OH), 3141(br, NH), 3002 (CH arom.), 2803 (CH aliph.), 2220 (CN), 1683 (C=O), 1549 (C=N), 1519 (C = C), 829 (*p*-substituted), 750 (*o*-substituted). MS: *m*/*z* (%) = 502 (M + 4, 0.77), 500 (M + 2, 1.35), 498 (M^+^, 2.15), 329 (9), 313 (9), 237 (10), 221 (20), 214 (10), 204 (9), 192 (32), 187 (47), 183 (22), 181 (22), 166 (22), 158 (15), 157 (29), 156 (33), 149 (82), 147 (51), 146 (41), 145 (33), 139 (52), 137 (67), 134 (27), 133 (67), 119 (50), 113 (46), 112 (89), 111 (100), 110 (91), 96 (78), 91 (58), 78 (45), 57 (36). ^1^H-NMR (DMSO-d_6_) *δ* ppm: 14.03 (1H, s, OH), 11.36 (1H, s, NH), 7.99–7.95 (3H, m, NH&2H_arom_), 7.69–7.67 (2H, d, *J* = 7.6 Hz, H_arom_), 7.52–7.50 (1H, d, *J* = 7.6 Hz, H_arom_), 7.32–7.25 (2H, m, H_arom_), 7.07–7.05 (1H, d, *J* = 7.6 Hz, H_arom_), 5.23 (2H, s, NCH_2_). Anal. Calcd for C_21_H_13_BrClN_5_O_3_, Calcd: C 50.57, H 2.63, N 14.04, Found: C 50.71, H 2.61, N 14.26.

#### *1*-*(2*-*chlorobenzyl)*-*8*-*hydroxy*-*6*-*(2*-*hydroxyphenyl)*-*2,4*-*dioxo*-*2,3,4,5*-*tetrahydro*-*1H*-*pyrimido[4,5*-*b][1, 4] diazepine*-*7*-*carbonitrile* (**5d**)

Yield: 51%, m.p. ≥ 300 °C. IR (ν_max_, cm^−1^) = 3617, 3495 (OH), 3154 (br, NH), 3025 (CH arom.), 2825 (CH aliph.), 2219 (CN), 1676, 1610 (C=O), 1548 (C=N), 1494 (C=C), 753 (*o*-substituted). MS: *m*/*z* (%) = 437 (M + 2, 0.3), 435 (M^+^, 0.9), 385 (37), 212 (28), 192 (37), 172 (49), 128 (29), 127 (100), 125 (79), 116 (19), 89 (41), 45 (15). ^1^H-NMR (DMSO-d_6_) *δ* ppm: 13.98 (1H, s, OH), 13.58 (1H, s, OH), 11.33 (1H, s, NH), 7.52–7.48 (3H, m, NH& 2H_arom_), 7.45–7.42 (2H, m, H_arom_), 7.35–7.24 (3H, m, H_arom_), 6.96–6.95 (1H, d, *J* = 7.6 Hz, H_arom_), 5.25 (2H, s, NCH_2_). Anal. Calcd for C_21_H_14_ClN_5_O_4_, Calcd.: C 57.87, H 3.24, N 16.07. Found: C 58.04, H 3.27, N 16.34.

#### *1*-*(2*-*chlorobenzyl)*-*8*-*hydroxy*-*6*-*(3*-*nitrophenyl)*-*2,4*-*dioxo*-*2,3,4,5*-*tetrahydro*-*1H*-*pyrimido[4,5*-*b][1, 4]diazepine*-*7*-*carbonitrile* (**5e**)

Yield: 49%, m.p. ≥ 300 °C. IR (ν_max_, cm^−1^) = 3619 (OH), 3154 (br, NH), 3027 (CH_arom_), 2822 (CH_aliph_), 2222 (CN), 1690, 1640 (C=O), 1578 (C=N), 1518, 1350 (NO_2_), 1466 (C=C), 808 (*m*-substituted), 752 (*o*-substituted). MS: *m*/*z* (%) = 466 (M + 2, 13), 464 (M^+^, 13.3), 460 (6), 439 (20), 361 (24), 299 (10), 298 (12), 259 (49), 257 (42), 240 (20), 183 (100), 124 (36), 97 (28), 85 (28), 57 (60), 40 (96). ^1^H-NMR (DMSO-d_6_) *δ* ppm: 14.36 (1H, s, OH), 11.42 (1H, s, NH), 8.90 (1H, s, H_arom_), 8.44–8.42 (1H, d, *J* = 8.0 Hz, H_arom_), 8.28–8.26 (1H, d, *J* = 8.0 Hz, H _arom_), 7.94 (1H, s, NH), 7.78–7.74 (1H, m, H_arom_), 7.52–7.50 (1H, d, *J* = 7.6 Hz, H_arom_), 7.32–7.25 (2H, m, H_arom_), 7.09–7.08 (1H, d, *J* = 7.6 Hz, H_arom_), 5.25 (2H, s, NCH_2_). Anal. Calcd for C_21_H_13_ClN_6_O_5_, Calcd.: C 54.26, H 2.82, N 18.08, Found: C 54.39, H 2.86, N 18.26.

#### *8*-*Amino*-*6*-*aryl*-*1*-*(2*-*chlorobenzyl)*-*2,4*-*dioxo*-*2,3,4,5*-*tetrahydro*-*1H*-*pyrimido[4,5*-*b] [1, 4]diazepine*-*7*-*carbonitriles* (**6a**–**d**)

A mixture of 5,6-diamino-1-(2-chlorobenzyl)uracil (**3**) (0.3 g, 1.12 mmol) and appropriate arylidene malononitrile (1.12 mmol) in DMF (3 ml) in presence of drops of TEA was heated under reflux for 6–7 h. The reaction mixture was evaporated under reduced pressure. The residue obtained was suspended in ethanol and filtered. The resulting solid was washed with ethanol and crystallized from DMF/ethanol (2:1).

#### *8*-*amino*-*1*-*(2*-*chlorobenzyl)*-*2,4*-*dioxo*-*6*-*phenyl*-*2,3,4,5*-*tetrahydro*-*1H*-*pyrimido[4,5*-*b] [1, 4] diazepine*-*7*-*carbonitrile* (**6a**)

Yield: 53%, m.p. ≥ 300 °C. IR (ν_max_, cm^−1^) = 3419, 3319 (NH_2_), 3190 (br, 2NH), 3061 (CH arom.), 2866 (CH aliph.), 2225 (CN), 1701, 1670 (C=O), 1560 (C=N), 1516 (C=C), 753 (*o*-substituted). MS: *m*/*z* (%) = 420 (M + 2, 0.6), 418 (M^+^, 2), 402 (9), 375 (11), 368 (44), 351 (11), 349 (9), 288 (12), 269 (14), 244 (16), 241 (21), 220 (100), 193 (15), 176 (19), 125 (36), 75 (35), 43 (28). ^1^H-NMR (DMSO-d_6_) *δ* ppm: 11.33 (1H, s, NH), 8.06–8.04 (1H, d, *J* = 6.8 Hz, H_arom_), 7.76 (2H, s, NH_2_), 7.75–7.42 (5H, m, NH & 4H_arom_), 7.32-7.26 (3H, m, H_arom_), 7.09–7.07 (1H, d, *J* = 6.4 Hz, H_arom_), 5.36 (2H, s, NCH_2_). Anal. Calcd for C_21_H_15_ClN_6_O_2_, Calcd.: C 60.22, H 3.61, N 20.07, Found: C 60.47, H 3.64, N 20.34

#### *8*-*amino*-*1*-*(2*-*chlorobenzyl)*-*6*-*(4*-*chlorophenyl)*-*2,4*-*dioxo*-*2,3,4,5*-*tetrahydro*-*1H*-*pyrimido[4,5*-*b][1, 4]diazepine*-*7*-*carbonitrile* (**6b**)

Yield: 69%, m.p. ≥ 300 °C. IR (ν_max_, cm^−1^) = 3435, 3333 (NH_2_), 3185 (br, NH), 3064 (CH arom.), 2822 (CH aliph.), 2220 (CN), 1707, 1664 (C=O), 1555 (C=N), 1497 (C=C), 815 (*p*-substituted), 753 (*o*-substituted). MS: *m*/*z* (%) = 457 (M + 4, 0.88), 455 (M + 2, 0.86), 453 (M^+^, 0.71), 401 (83), 358 (9), 351 (9), 241 (8), 228 (9), 217 (7), 202 (8), 184 (18), 182 (17), 180 (14), 148 (14), 140 (18), 139 (14), 138 (11), 134 (41), 127 (21), 125 (64), 124 (67), 99 (21), 89 (68), 73 (43), 63 (25), 44 (60), 42 (18), 40 (100). ^1^H-NMR (DMSO-d_6_) *δ* ppm: 11.37 (1H, s, NH), 7.77 (2H, s, NH_2_), 7.52–7.48 (3H, m, NH&2H_arom_), 7.36–7.26 (5H, m, H _arom_), 7.09–7.07 (1H, d, H_arom_), 5.36 (2H, s, NCH_2_). Anal. Calcd for C_21_H_14_Cl_2_N_6_O_2_, Calcd.: C 55.64, H 3.11, N 18.54, Found: C 55.82, H 3.17, N 18.69

#### *8*-*amino*-*6*-*(4*-*bromophenyl)*-*1*-*(2*-*chlorobenzyl)*-*2,4*-*dioxo*-*2,3,4,5*-*tetrahydro*-*1H*-*pyrimido[4,5*-*b][1, 4]diazepine*-*7*-*carbonitrile* (**6c**)

Yield: 64%, m.p. ≥ 300 °C. IR (ν_max_, cm^−1^) = 3312 (NH_2_), 3144 (br, NH), 3085 (CH arom.), 2973, 2801 (CH aliph.), 2218 (CN), 1687, 1648 (C=O), 1550 (C=N), 1519 (C=C), 830 (*p*-substituted), 752 (*o*-substituted). MS: *m*/*z* (%) = 501 (M + 4, 0.11), 499 (M + 2, 0.11), 497 (M^+^, 0.12), 368 (3), 211 (7), 185 (9), 183 (31), 155 (10), 129 (19), 127 (7), 125 (19), 123 (9), 109 (14), 107 (8), 98 (19), 85 (32), 83 (24), 73 (100), 71 (41), 57 (18), 43 (54). ^1^H-NMR (DMSO-d_6_) *δ* ppm: 11.37 (1H, s, NH), 8.00–7.99 (1H, d, *J* = 7.6 Hz, H_arom_), 7.94–7.92 (1H, d, *J* = 7.6 Hz, H_arom_), 7.71–7.69 (1H, m, H_arom_), 7.67–7.24 (7H, m, NH_2_ & NH & 4H_arom_.), 7.05–7.03 (1H, d, *J* = 7.6 Hz, H_arom_), 5.24 (2H, s, NCH_2_). Anal. Calcd for C_21_H_14_BrClN_6_O_2_, Calcd.: C 50.67, H 2.84, N 16.88, Found: C 50.84, H 2.89, N 16.98

#### *8*-*amino*-*1*-*(2*-*chlorobenzyl)*-*6*-*(2*-*hydroxyphenyl)*-*2,4*-*dioxo*-*2,3,4,5*-*tetrahydro*-*1H*-*pyrimido[4,5*-*b][1, 4]diazepine*-*7*-*carbonitrile* (**6d**)

Yield: 54%, m.p. ≥ 300 °C. IR (ν_max_, cm^−1^) = 3618 (OH), 3420, 3349 (NH_2_), 3195 (br, NH), 3060 (CH arom.), 2967, 2835 (CH aliph.), 2214 (CN), 1695, 1650 (C=O), 1555 (C=N), 1510 (C=C), 755 (*o*-substituted). MS: *m*/*z* (%) = 436 (M + 2, 0.23), 434 (M^+^, 0.64), 366 (20), 333 (11), 300 (14), 193 (13), 166 (8), 165 (16), 164 (12), 127 (33), 125 (100), 94 (24), 91 (25), 90 (11), 89 (39). ^1^H-NMR (DMSO-d_6_) *δ* ppm: 13.72 (1H, s, OH), 11.36 (1H, s, NH), 7.95–7.94 (1H, d, *J* = 7.6 Hz, H_arom_), 7.54–7.44 (4H, m, NH_2_ & NH & H_arom_), 7.27–7.23 (4H, m, H_arom_), 7.22-7.20 (2H, d, *J* = 7.6 Hz, H_arom_), 5.31 (2H, s, NCH_2_). Anal. Calcd for C_21_H_15_ClN_6_O_3_, Calcd.: C 58.00, H 3.48, N 19.33, Found: C 58.26, H 3.54, N 19.57

#### *7*-*Aryl*-*1*-*(2*-*chlorobenzyl)pteridine*-*2,4(1H,3H)*-*diones* (**7a**–**d**)

A mixture of 5,6-diamino-1-(2-chlorobenzyl)uracil (**3**) (0.3 g, 1.12 mmol) and appropriate phenacyl bromide (1.12 mmol) in DMF (3 ml) in presence of drops of TEA was heated under reflux for 2–3 h. After cooling, ethanol was added, the formed crystals were collected by filtration, washed with ethanol and crystallized from ethanol.

#### *1*-*(2*-*chlorobenzyl)*-*7*-*phenylpteridine*-*2,4(1H,3H)*-*dione* (**7a**)

Yield: 71%, m.p. ≥ 300 °C. IR (ν_max_, cm^−1^) = 3169 (NH), 3030 (CH arom.), 2842 (CH aliph.), 1724, 1693 (C=O), 1536 (C=C), 752 (*o*-substituted), 715, 680 (monosubstituted benzene ring). MS: *m*/*z* (%) = 366 (M^+^+2, 1), 364 (M^+^, 1), 350 (9), 345 (32), 336 (10), 264 (17), 252 (12), 228 (27), 216 (19), 186 (56), 185 (100), 184 (28), 173 (44), 172 (22), 159 (75), 158 (15), 91 (75). ^1^H-NMR (DMSO-d_6_) *δ* ppm: 12.07 (1H, s, NH), 9.20 (1H, s, CH-6), 8.07–8.05 (2H, d, *J* = 9.6 Hz, H_arom_), 7.54–7.49 (4H, m, H _arom_), 7.30–7.19 (3H, m, H_arom_), 5.46 (2H, s, NCH_2_). Anal. Calcd for C_19_H_13_ClN_4_O_2_, Calcd.: C 62.56, H 3.59, N 15.36, Found: C 62.73, H 3.61, N 15.49

#### *1*-*(2*-*chlorobenzyl)*-*7*-*(4*-*methoxyphenyl)pteridine*-*2,4(1H,3H)*-*dione* (**7b**)

Yield: 74%, m.p. ≥ 300 °C. IR (ν_max_, cm^−1^) = 3174 (NH), 3053 (CH arom.), 2966, 2832 (CH aliph.), 1718, 1680 (C=O), 1529 (C=C), 846 (*p*-substituted), 748 (*o*-substituted). MS: *m*/*z* (%) = 396 (M + 2, 2.5), 394 (M^+^, 7), 360 (25), 359 (100), 288 (8), 179 (7), 158 (7), 127 (17), 125 (54), 89 (25). ^1^H-NMR (DMSO-d_6_) *δ* ppm: 12.00 (1H, s, NH), 9.14 (1H, s, CH-6), 8.06–8.04 (2H, d, *J* = 8.8 Hz, H_arom_), 7.53–7.51 (1H, d, *J* = 9.2 Hz, H_arom_), 7.29–7.18 (3H, m, H _arom_), 7.06–7.04 (2H, d, *J* = 8.8 Hz, H_arom_), 5.44 (2H, s, NCH_2_), 3.82 (3H, s, CH_3_). ^13^C-NMR (DMSO-d_6_) *δ* ppm: 162.0, 159.9, 152.9, 150.3, 148.1, 136.3, 134.0, 131.3, 129.3, 129.2, 128.6, 127.3, 126.6, 126.5, 114.7, 55.5, 42.2. Anal. Calcd for C_20_H_15_ClN_4_O_3_, Calcd.: C 60.84, H 3.83, N 14.19, Found: C 60.98, H 3.80, N 14.34

#### *1*-*(2*-*chlorobenzyl)*-*6,7*-*diphenylpteridine*-*2,4(1H,3H)*-*dione* (**7c**)

Yield: 68%, m.p. ≥ 300 °C. IR (ν_max_, cm^−1^) = 3150 (NH), 3022 (CH arom.), 2823 (CH aliph.), 1725, 1687 (C=O), 1522 (C=C), 752, 696 (phenyl group), 752 (*o*-substituted). MS: *m*/*z* (%) = 442 (M + 2, 0.10), 440 (M^+^, 0.10), 318 (12), 317 (57), 127 (31), 126 (8), 125 (100), 104 (17), 89 (24), 77 (11). ^1^H-NMR (DMSO-d_6_) *δ* ppm: 11.33 (s, 1H, NH), 8.05–8.04 (2H, d, *J* = 6.8 Hz, H_arom_), 7.52–7.45 (5H, m, H_arom_), 7.36–7.23 (6H, m, H_arom_), 7.08–7.06 (1H, d, *J* = 7.6 Hz, H_arom_), 5.24 (s, 2H, NCH_2_). Anal. Calcd for C_25_H_17_ClN_4_O_2_, Calcd.: C 68.11, H 3.89, N 12.71, Found: C 68.24, H 3.95, N 12.87.

#### *1*-*(2*-*chlorobenzyl)*-*7*-*(4*-*nitrophenyl)pteridine*-*2,4(1H,3H)*-*dione* (**7d**)

Yield: 58%, m.p. ≥ 300 °C. IR (ν_max_, cm^−1^) = 3100 (NH), 3040 (CH arom.), 2964 (CH aliph.), 1738, 1647 (C=O), 1548 (C=C), 1515, 1368 (NO_2_), 869 (*p*-substituted), 746 (*o*-substituted). MS: *m*/*z* (%) = 411 (M^+^+2, 0.77), 409 (M^+^, 3.35), 376 (31), 299 (20), 255 (98), 236 (29), 212 (17), 187 (34), 172 (17), 159 (21), 157 (35), 146 (23), 124 (100), 71 (29). ^1^H-NMR (DMSO-d_6_) *δ* ppm: 12.15 (1H, s, NH), 9.32 (1H, s, CH-6), 8.34–8.32 (2H, d, H_arom_), 7.52–7.26 (6H, m, H_arom_.), 5.48 (2H, s, NCH_2_). Anal. Calcd for C_19_H_12_ClN_5_O_4_, Calcd.: C 55.69, H 2.95, N 17.09, Found: C 55.87, H 2.97, N 17.41

#### *4*-*(2*-*chlorobenzyl)*-*3H*-*[1,2,3] triazolo[4,5*-*d]pyrimidine*-*5,7(4H,6H)*-*dione* (**8**)

A mixture of 5,6-diamino-1-(2-chlorobenzyl)uracil (**3**) (0.3 g, 1.12 mmol), was dissolved in conc. HCl (4 ml) and sodium nitrite (1.12 mmol) in water (1.5 ml) was stirred at room temperature for 2 h. The formed yellowish white precipitate was filtered, washed with ethanol and crystallized from DMF/ethanol (1:2).

Yield: 78%, m.p. ≥ 300 °C. IR (ν_max_, cm^−1^) = 3358, 3182 (NH), 3061 (CH_arom_), 2844 (CH aliph.), 1721, 1672 (C=O), 1582 (C=N), 1467 (C=C), 748 (*o*-substituted). MS: *m*/*z* (%) = 279 (M^+^+2, 0.89), 277 (M^+^, 1.28), 276 (3.56), 259 (11), 243 (25), 241 (82), 214 (19), 199 (25), 127 (87), 125 (100), 116 (14). ^1^H-NMR (DMSO-d_6_) *δ* ppm: 15.76 (1H, s, NH), 11.61 (1H, s, NH), 7.51–7.49 (1H, d, *J* = 9.2 Hz, H_arom_), 7.32–7.23 (2H, m, H_arom_), 7.16–7.14 (1H, d, *J* = 9.2 Hz, H_arom_), 5.14 (2H, s, NCH_2_). ^13^C-NMR (DMSO-d_6_) *δ* ppm: 156.5, 150.8, 149.9, 133.1, 131.5, 129.4, 129.0, 128.6, 127.4, 127.3, 44.3. Anal. Calcd for C_11_H_8_ClN_5_O_2_, Calcd.: C 47.58, H 2.90, N 25.22, Found: C 47.69, H 2.89, N 25.45.

#### *3*-*(2*-*chlorobenzyl)*-*8*-*methyl*-*3,9*-*dihydro*-*1H*-*purine*-*2,6*-*dione* (**9**)

A mixture of 5,6-diamino-1-(2-chlorobenzyl)uracil (**3**) (0.3 g, 1.12 mmol), acetic anhydride (1.5 ml) and acetic acid (5 ml) was heated under reflux for 8 h. After cooling, the brown precipitate was collected by filtration, washed with ethanol and crystallized from DMF/ethanol (1:1).

Yield: 72%, m.p. ≥ 300 °C. IR (ν_max_, cm^−1^) = 3149, 3120 (2NH), 3024 (CH arom.), 2807 (CH aliph.), 1691, 1660 (C=O), 1566 (C=N), 1509 (C=C), 746 (*o*-substituted). MS: *m*/*z* (%) = 292 (M+2, 1.65), 290 (M^+^, 4), 256 (15), 255 (100), 127 (23), 125 (70), 89 (14). ^1^H-NMR (DMSO-d_6_) *δ* ppm: 13.19 (1H, s, NH), 11.15 (1H, s, NH), 7.50–7.48 (1H, d, *J* = 9.2 Hz, H_arom_), 7.30–7.24 (2H, m, H_arom_), 6.93–6.90 (1H, d, *J* = 9.2 Hz, H_arom_), 5.13 (2H, s, NCH_2_), 2.31 (3H, s, CH_3_). ^13^C-NMR (DMSO-d_6_) *δ* ppm: 154.3, 150.9, 150.6, 149.3, 134.0, 131.3, 129.3, 128.7, 127.4, 126.6, 106.7, 43.0, 14.2. Anal. Calcd for C_13_H_11_ClN_4_O_2_, Calcd.: C 53.71, H 3.81, N 19.27, Found: C 53.94, H 3.87, N 19.43

#### *[3*-*(2*-*chlorobenzyl)*-*2,6*-*dioxo*-*2,3,6,9*-*tetrahydro*-*1H*-*purin*-*8*-*yl]acetonitrile* (**10**)

A mixture of 5,6-diamino-1-(2-chlorobenzyl)uracil (**3**) (0.3 g, 1.12 mmol) and malononitrile (1.12 mmol) was heated for 10 min without solvent. The residue was treated with ethanol; the formed precipitate was filtered, and washed with ethanol and crystallized from DMF into colourless crystals.

Yield: 77%, m.p. ≥ 300 °C. IR (ν_max_, cm^−1^) = 3328, 3175 (NH), 3082 (CH arom.), 2925 (CH aliph.), 2200 (CN), 1660, 1616 (C=O), 1549 (C=N), 1510 (C=C), 752 (*o*-substituted). MS: *m*/*z* (%) = 317 (M^+^+2, 0.6), 315 (M^+^, 1), 274 (4), 264 (5), 253 (5), 242 (25), 241 (10), 225 (10), 213 (12), 193 (7), 186 (25), 185 (35), 168 (12), 164 (17), 127 (16), 125 (100), 123 (17). ^1^H-NMR (DMSO-d_6_) *δ* ppm: 12.49 (1H, s, NH), 10.51 (1H, s, NH), 7.47–7.29 (4H, m, H_arom_), 5.08 (2H, s, NCH_2_), 4.10 (2H, s, CH_2_CN). Anal. Calcd for C_14_H_10_ClN_5_O_2_, Calcd.: C 53.26, H 3.19, N 22.18, Found: C 53.41, H 3.17, N 22.39.

### Biological activity assay

#### Antimicrobial activity assay

The antimicrobial activity was measured using two different agar diffusion methods; paper-disk and agar-well diffusion methods. Samples were dissolved in DMSO. Aliquots of 20 µl (conc. 50 mg/ml) were soaked on filter paper disks (5 mm diameter, Wattman no. 1) and left to dry under aseptic conditions for 1 h. Paper-disk diffusion assay [35] with some modifications has been followed to measure the antimicrobial activity. Twenty milliliters of medium seeded with test organisms were poured into 9 cm sterile Petri dishes. After solidification, the paper disks were placed on the inoculated agar plates and allowed to diffuse the loaded substances into refrigerator at 4 °C for 2 h to allow the diffusion of substances. The plates were incubated for 24 h at 35 °C. Both bacteria and yeasts were grown on nutrient agar medium (g/l): Beef extract, 3; peptone, 10; and agar, 20. The pH was adjusted to 7.2. Fungal strain was grown on potato dextrose agar medium (g/l): Potato extract, 4; Dextrose, 20; Agar No. 1 15 (pH 6). The diameter of inhibition zone was measured. In the agar-well diffusion method [36], cups (5 mm in diameter), were cut using a sterile cork borer and the agar discs were removed. Cups were filled with 20 μl of samples. Benzylpenicillin and Nystatin were used as antibacterial and antifungal control, respectively. After incubation, the diameter of inhibition zones was measured against a wide range of test microorganisms comprising: Gram positive bacteria; (*B. subtilis* ATCC6633 and *S. aureus* ATCC6538-P), Gram negative bacteria (*P. aeruginosa* ATCC 27853), yeasts (*C. albicans* ATCC 10231 and *S. cerevisiae* ATCC 9080) and the fungus *A. niger* NRRL A-326. Minimal inhibition concentrations (MIC) of the active compounds have been determined using disk diffusion method according to methods described in [37, 38]. Tenth fold dilutions of starting concentration had been done to make different concentrations.

#### Antioxidant activity assay

The percentage of antioxidant activity (AA%) was measured using DPPH free radical assay as described by [39]. The samples were reacted with DPPH (2,2-diphenyl-1-picryl-hydrazyl-hydrate) in DMSO solution. The reaction mixture consisted of 50 µl (conc. 2.5 mg/ml) of each sample, 3 ml of 0.5 mM DPPH/DMSO solution. The reduction of DPPH by antioxidant compounds changes the color from deep violet into light yellow. The absorbance was read at 517 nm after 60 min of reaction using a UV–Vis spectrophotometer (Shimadzu). The mixture of DMSO (3 ml) and sample (50 µl) serve as blank. The control is 3 ml of prepared DPPH solution (0.5 mM). The scavenging activity percentage (AA%) was calculated according to Ref. [40].

## Conclusions

A series of newly synthesized compounds of pyrimido[4,5-*b*][1, 4]diazepines **5a**–**e, 6a**–**d**, lumazines **7a**–**d**, triazolo[4,5-*d*]pyrimidine **8** and xanthines **9**, **10** were prepared by a simple method from 5,6-diamino-1-(2-chlorobenzyl)uracil **3**. The novel compounds were screened for both antimicrobial and antioxidant activities. Compounds **5a**, **5b**, **6a**, **6d** and **8** showed a wide range activity against the pathogenic tested microbes (*S. aureus*, *B. subtilis*, *P. aeruginosa*, *C*. *albicans*, and *S. cerevisiae*) in comparison to the standard drug Benzylpenicillin. Compound **8** was the only novel synthesized compound exhibited activity against the fungus *A. niger* in comparison to the standard drug Nystatin. On the other hand, Compound **5a** showed the highest antioxidant activity followed by compound **8**. While, compounds **7a** and **7b** showed no antioxidant activity.
