# Erratum to: One pot synthesis, antimicrobial and antioxidant activities of fused uracils: pyrimidodiazepines, lumazines, triazolouracil and xanthines

**DOI:** 10.1186/s13065-017-0302-4

**Published:** 2017-08-01

**Authors:** Samar A. El-Kalyoubi, Eman A. Fayed, Ahmed S. Abdel-Razek

**Affiliations:** 1 Department of Pharmaceutical Organic Chemistry, Faculty of Pharmacy (Girls), Al-Azhar University, Nasr City, Cairo, 11651 Egypt; 2 Medical Chemistry Department, Faculty of Medicine (Female Section), Jazan University, Jazan, 45142 Saudi Arabia; 3 Department of Microbial Chemistry, Genetic Engineering and Biotechnology Division, National Research Centre, El-Buhouth St., Dokki-Cairo, 12622 Egypt

## Erratum to: Chemistry Central Journal (2017) 11:66 DOI 10.1186/s13065-017-0294-0

After the publication of this work [1] it was noticed that the author’s name was spelt incorrectly. The author’s name should be spelt as: Ahmed S. Abdel-Razek.

The original article was corrected.
